# Supplementary figures and images for: Nerve regeneration using a Bio 3D conduit derived from umbilical cord–Derived mesenchymal stem cells in a rat sciatic nerve defect model
Source: PLoS One. 2024 Dec 23;19(12):e0310711. doi: 10.1371/journal.pone.0310711 (PMC11666056; doi:10.1371/journal.pone.0310711)

## Slide 1
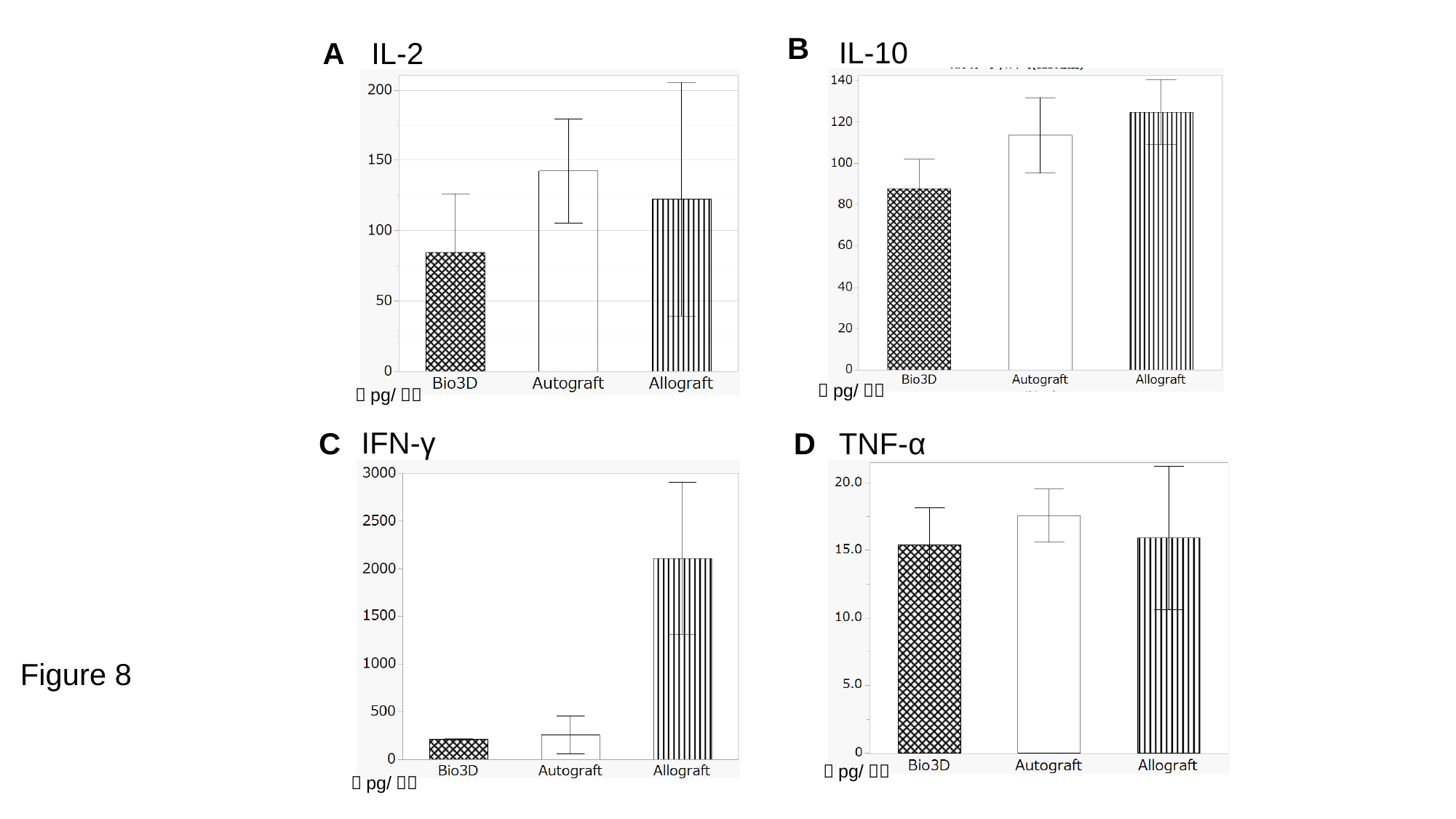

B
IL-10
A
IL-2
（pg/㎖）
（pg/㎖）
IFN-γ
C
D
TNF-α
Figure 8
（pg/㎖）
（pg/㎖）

Supplement: S1 File — There was no significant difference in cytokine concentrations among the three groups. (PPTX) [file pone.0310711.s001.pptx]
